# A Treatise on the Principles of Pharmacy, Etc.

**Published:** 1894-02

**Authors:** 


A Treatise on the Principles of Pharmacy and Pharmaceutical Chemistry, Including a Laboratory Guide. By James Kennedy, Ph. G., M. D., Professor of Pharmacy and Botany, and Dean of the Faculty of the Department of Pharmacy of the University of Texas; Delegate-elect to Revision Committee of U. S. Pharmacopœia; Ex-Vice-President Texas State Pharmaceutical Association; Ex-President of the San Antonio Pharmaceutical Association; President of the Galveston Pharmaceutical Association; Member of the Louisville College of Pharmacy, etc., etc., etc., and Seth M. Morris, M. D., B. Sc., Professor of Chemistry and Physics and Secretary of the Faculty in the School of Pharmacy of the University of Texas; Professor of Chemistry and Toxicology in the Medical Department of the University of Texas; Ex-Assistant to the Chair in the University of Texas; President of the Alumni of the University of Texas, etc., etc., etc.

Drs. Kennedy and Morris have in preparation and will issue early this summer or fall a work as above outlined. Its advent will be looked for with interest by the profession and no doubt it will be very popular. The authors will say in their preface, which we are allowed to copy in advance of their publication:

"Whenever a new book makes its appearance, it is only reasonable to expect that it shall be complete in all its parts, and shall be adorned in a manner that custom has sanctioned as the proper one; hence in our preface we shall make the usual apologies for having added our modest tribute to the vast number of good books already extant and endeavor to persuade our readers that this work of ours is destined to fill a long felt want, and that it is the product or outgrowth of an existing necessity for a book of this character.

"When the author, who is in charge of the Department of Pharmacy of the University, first assumed the task of instruction in this department, he sorely felt the need of some systematic scheme for use in the Pharmaceutical Laboratory. It is his opinion that up to the time of going to press, no work is produced that fills in a satisfactory manner the functions of a Laboratory Guide in Pharmacy.

"There are a great number of laboratory guides for the student of chemistry, and several of them leave but little to be desired. It was the design of the authors in the beginning to publish a Laboratory Guide in Pharmacy, but when begun they were impressed with the opportunity for enlarging the scope and usefulness of their undertaking, and have enlarged the work to such an extent as make the Laboratory Guide an appendix. We do not wish to be understood as saying that the importance of the Guide has been lessened by so doing; to the contrary, we have introduced much of the additional matter for the express purpose of elaborating the Guide and making its language more clear and explicit. This book has been designed with a view to utility to the student, the teacher and the practical pharmacist, and each portion of the book has received its full share of attention.

"Part I embraces chemical and pharmaceutical definitions, the chemical elements, properties of matter, chemical philosophy, chemical analysis, metrology, physics of pharmacy, including the phenomena of solution, extraction, filtration, vaporization, precipitation, application, control and measurement of heat, etc.

"Part II. embraces the organic and inorganic medicines and pharmaceuticals, describing their physical, chemical and medical properties and doses, and, in the case of vegetable drugs, their habitat and botanical characters. Those drugs of animal origin are described in detail, including the source from which they are obtained, tests of identity, purity and strength are given whenever practicable.

"A chapter descriptive of the general properties of preparations, giving in a general way the resemblances and differences of fluid extracts, tinctures, etc., and describing their method of manufacture, is included.

"Part III. is devoted to the Laboratory Guide, both pharmaceutical and chemical, and the results to be obtained by following this order of experiments are described and fully explained in the chapters referred to in that part.

"Part IV. contains chapters on urine analysis, water analysis, plant analysis, food analysis, toxicology, active principles, oils, fats, and the new remedies.

"A formulary of unofficial preparations, tables of comparative weights, of relations between the specific gravities of solutions and their strengths, etc., are appended.

"The plan followed throughout the work is to give explanations of the phenomena, herein described, in as clear and concise a manner as possible, thus enabling the student to trace a given effect to its cause. We do not deem it expedient or wise to merely state, as is done in any text books, that if such and such substances are mixed that such and such a reaction will occur, without entering into an explanation as to the reasons therefor. It is our belief that unless the student is made to comprehend why the experiment is performed and the phenomena concerned in the production of a result, the subject can not be understood by him. The general plan adopted by us is to begin the study of our subject with a thorough consideration of the fundamental principles of the sciences treated of herein, and to gradually lead the student up to the advanced knowledge of the subject. Our aim has been to closely link related facts and illustrate their interdependence. How well we have succeeded in our undertaking, we must leave to the profession to determine.''

